# Synchronous Acute Appendicitis and Cholecystitis in a Paediatric Patient with Salmonella Enteritis

**DOI:** 10.7759/cureus.7214

**Published:** 2020-03-08

**Authors:** Christo T Joseph, Chi Lap Nicholas Tsang, David Goltsman, Natalia L Garibotto, Allan Mekisic

**Affiliations:** 1 General Surgery, The Wollongong Hospital, Wollongong, AUS; 2 Surgery, The Wollongong Hospital, Wollongong, AUS; 3 Breast and Endocrine Surgery, Illawarra Shoalhaven Local Health District, Wollongong, AUS

**Keywords:** appendicitis, cholecystitis, salmonella, appendicectomy, cholecystectomy, paediatric, synchronous

## Abstract

We present the case of a 13-year-old male who presented with right upper quadrant pain and diarrhoea after recently travelling from Bali, Indonesia. He had a normal white cell count of 8x10^9/L and elevated c-reactive protein (CRP) of 205 mg/L with normal liver function tests. Originally thought to be appendicitis, given the rarity of cholecystitis in a child, he was commenced on broad-spectrum antibiotics and was taken to the operating theatre based on his clinical presentation. Diagnostic laparoscopy revealed inflammatory change in both the gallbladder and appendix and a laparoscopic cholecystectomy and appendicectomy were performed simultaneously. Histopathology results confirmed cholecystitis and appendicitis and a stool culture confirmed the presence of Salmonella serotype B. Synchronous cholecystitis and appendicitis is an exceedingly rare phenomenon with only a handful of cases reported in the literature. This is the first case in the literature of this phenomenon occurring in the paediatric population; surgeons need to be aware of this rare possibility even in the paediatric population and especially in those patients with an atypical presentation or in recent travellers experiencing gastroenteritis.

## Introduction

Synchronous appendicitis and cholecystitis is a rare phenomenon in the current literature and this is the first case reported in a paediatric patient. A recent review by Shweiki et al. theorized that the condition is actually under recognized based on higher incidence reported in the pre-antibiotic era (circa early 1900s) and that antibiotic treatment of appendicitis has drastically reduced the detection of synchronous gallbladder disease [1]. The theory is that the mechanism of synchronous infection could be due to pathogen predilection for the appendix and gallbladder, the promotion of cholestasis and bacterial translocation, or invasion of the portal venous system from the appendix [1]. Furthermore, hyperbilirubinaemia has been observed in acute appendicitis and it is thought to be due to impaired bilirubin excretion secondary to bacterial translation to the portal system [2]. From a technical perspective, a single staged combined procedure has been demonstrated to be effective in treating this pathology [2,3].

## Case presentation

**Figure 1:**
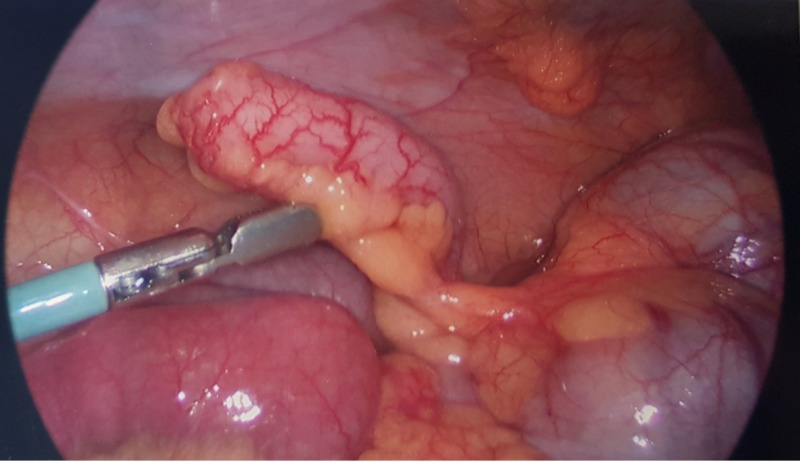
Inflamed appendix seen on laparoscopy

**Figure 2:**
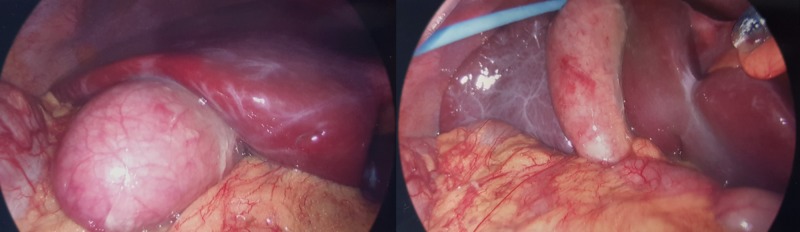
Inflamed and oedematous gallbladder noted on laparoscopy

A 13-year-old male was brought to the emergency department with a four-day history of fever, progressively worsening right-sided abdominal pain as well as diarrhoea and vomiting. Further history revealed that he had recently travelled to Bali, Indonesia, but there were no sick contacts in the family. General examination revealed an unwell child who was tachycardic and febrile. Abdominal examination revealed a positive Murphy’s sign with peritonism of the right upper quadrant and right lower quadrant. Pathology tests showed a white cell count of 8x10^9/L and a raised c-reactive protein (CRP) level of 205mg/L but normal liver function tests. Based on this clinical information, the decision was made for a diagnostic laparoscopy. Diagnostic laparoscopy was conducted with a standard Hasson umbilical entry and ports inserted in preparation for an appendicectomy. Inspection of the intra-abdominal cavity revealed synchronous cholecystitis and appendicitis macroscopically (Figures 1-2).

Firstly, a laparoscopic appendicectomy was performed which was then followed by a laparoscopic cholecystectomy using Strasberg dissection of the gallbladder. An intraoperative cholangiogram was unremarkable and showed free flow of contrast into the duodenum. Acute appendicitis and acute acalculous cholecystitis were confirmed on histopathology. Post-operatively, the patient remained generally unwell and lethargic with ongoing diarrhoea, with CRP rising to 306. The paediatric infectious diseases team was consulted and the patient was empirically treated with meropenem and azithromycin due to the possibility of extended spectrum beta-lactamase (ESBL), salmonella or campylobacter. A stool test was positive for Salmonella serogroup B, which was pan-sensitive and his antibiotics were rationalised. He clinically improved over the next few days with down-trending inflammatory markers and he was discharged home on day seven post-operatively with a course of oral azithromycin.

## Discussion

Synchronous appendicitis and cholecystitis is a rare phenomenon and this is the first case reported in a paediatric patient. It has been proposed that the condition is actually under recognized based on higher incidence reported in the pre-antibiotic era and the hypothesis that antibiotic treatment of appendicitis has drastically reduced the development of synchronous gallbladder disease [1]. It has been theorised that the mechanism of synchronous infection could be due to pathogen predilection for the appendix and gallbladder, the promotion of cholestasis in sepsis and bacterial translocation, or invasion of the portal venous system from the appendix [1]. Salmonella infection is a known rare cause of acalculous cholecystitis in the paediatric population but a Salmonella serotype B infection causing synchronous appendicitis and cholecystitis has not yet been described [3]. From a technical perspective, a single staged combined procedure has been demonstrated to be effective in treating this dual pathology as seen in this case [2]. Lee et al. have also described successful non-operative management of this dual pathology with antibiotics and this is an option that can be considered by clinicians especially in those patients who may not be fit for surgery [4].

Synchronous appendicitis and cholecystitis can be quite challenging to diagnose due to the variable clinical presentations as patients have presented with either right upper quadrant pain, diffuse right-sided pain or right iliac fossa pain as noted in the literature [5]. In the adult population, computed tomography (CT) of the abdomen is the most reliable diagnostic imaging modality used, along with abdominal ultrasound, to confirm cholecystitis [5]. In the paediatric population, clinical history and examination can be supplemented with biochemistry and diagnostic imaging such as an abdominal ultrasound. As this case report shows, it is important to consider dual pathology whilst conducting diagnostic laparoscopy especially in those patients with atypical presentations.

## Conclusions

Synchronous appendicitis and cholecystitis is an exceedingly rare phenomenon in the literature. This case report is the first instance in the literature of synchronous acute cholecystitis and appendicitis in a paediatric patient with Salmonella enteritis. Clinicians should be aware of this possibility especially in those patients with an atypical presentation or in recent travellers experiencing gastroenteritis.
